# Impact of anti‐obesity medication initiation and duration on weight loss in a comprehensive weight loss programme

**DOI:** 10.1002/osp4.361

**Published:** 2019-08-12

**Authors:** R. Safavi, A. Lih, S. Kirkpatrick, S. Haller, M. R. Bailony

**Affiliations:** ^1^ University of California Santa Cruz California USA; ^2^ Research Department Enara Health Inc. San Mateo California USA

**Keywords:** obesity treatment, weight loss, weight management programme, weight‐reducing drugs

## Abstract

**Objective:**

This retrospective study aimed to evaluate the impact of anti‐obesity medication (AOM) initiation, usage and duration on weight loss in a 72‐week precision obesity programme. The type of AOM, diet and exercise plan was chosen based upon an individual's biological and psychosocial needs. The 72‐week study duration allowed for a fair investigation of the downstream impact of delayed versus early AOM initiation.

**Methods:**

Participants, aged ≥18 years with body mass index ≥30 kg m^−2^, enrolled from 1 March 2015 to 1 April 2017, were included. Subgroups were assigned by AOM usage (users versus non‐users, early [before 8 weeks] versus delayed [after 8 weeks] AOM initiation and short [<6 months] versus long [≥6 months] AOM duration). Primary endpoints included change in baseline weight at 72 weeks and proportions achieving ≥5%, ≥10% and ≥15% weight loss. Outcomes were compared between subgroups.

**Results:**

Mean age and body mass index (*N* = 129) were 45.0 ± 14.0 years and 37.0 ± 6.0 kg m^−2^, respectively; 67% were female. At week 72, AOM users (*N* = 71) achieved significantly greater mean percentage reduction in baseline weight than non‐users (*N* = 58). On average, baseline weight decreased by 14.04 ± 6.2% in users versus 10.9 ± 6.8% in non‐users (*P* = 0.008); 84% and 94% of non‐user and AOM users lost >5% weight loss (*P* = 0.006). A higher proportion of users lost ≥15% of weight (45.1% vs. 19.0%; *P* < 0.001). Mean percentage reduction in weight was greater for early versus delayed starters (−17.60 ± 5.3% vs. −13.95 ± 5.5%; *P* = 0.024), and longer AOM usage trended towards increased weight loss.

**Conclusion:**

Early initiation of AOM may enhance weight loss.

## Introduction

Recent guidelines generally recommend anti‐obesity medications (AOMs) only for patients who have failed to achieve 5% weight loss through dietary, exercise and/or lifestyle modification interventions alone 1, 2, 3, 4, 5. However, the optimal time to introduce an AOM in the setting of a comprehensive weight loss programme has not been well studied 6. Should physicians always start with diet and exercise before adding a medication or should they initiate both together? Does the intensity of diet impact the initiation and efficacy of AOM? How important is medication duration and compliance? As the obesity epidemic continues to grow, the different combinations and order in which therapies are delivered deserve more attention and study 7.

Recently, a study of 150 adults showed that adding liraglutide of 3.0 mg to intensive behavioural therapy (IBT) for obesity at programme onset showed larger weight loss in the IBT‐liraglutide group (11.5 ± 1.3%) than the IBT‐alone group (6.1 ± 1.3%) but that meal replacement did not increase weight loss when added to IBT and liraglutide (11.8 ± 1.3%) 8. This differs from a study that showed that of sibutramine, lifestyle modification and a portion‐controlled diet produced significantly greater weight loss (16.5 ± 8.0%) than both the sibutramine alone (4.1 ± 6.3%) and the sibutramine plus lifestyle therapy (10.8 ± 10.3%) cohorts 9. These studies suggest that early initiation of medication improves results of lifestyle therapy while dietary intensity may enhance the efficacy of some obesity medications but not others. Restricting the use of AOMs to patients who demonstrate lifestyle failure may not be in best interests of patients when the sum may be greater than the parts.

On the contrary, there is also evidence suggesting that adding AOM only after patients achieve 5% or greater weight loss may produce better long‐term weight loss and maintenance. In one study, 561 patients (mean body mass index [BMI], 34.8 kg m^−2^) were randomized to either of two topiramate (TPM) regimens or placebo after losing ≥8% of their initial body weight following an 8‐week low‐calorie diet (LCD) 10. At week 44 (including the 8‐week LCD run‐in), a modified intent‐to‐treat analysis showed that TPM significantly reduced weight by 15.4% and 16.5% in the 96 and 192 mg d^−1^ arms, respectively, compared with 8.9% in the placebo arm 10. In contrast, TPM randomization from the onset of a weight loss programme has been associated with a 5–6.5% reduction in baseline weight at 52 weeks 11. Similarly, in the SCALE Maintenance trial, patients (*N* = 212) who initiated liraglutide of 3.0 mg d^−1^ after losing 6% of body weight during a 4‐ to 12‐week LCD run‐in achieved 12.2% weight loss (6.2% from randomization) at 56 weeks compared with 6% weight loss in the placebo arm (*N* = 210) 12. On the other hand, patients randomized to liraglutide at the programme onset achieved only 8% weight loss at 56 weeks 13. While not comparative, these studies collectively suggest that delaying the start of AOM in the context of a comprehensive weight loss programme is not only beneficial for maintaining weight loss but may also increase the total weight loss achieved. The potential for additive weight loss with more precise timing of pharmacotherapy, therefore, warrants further investigation of the optimal time for initiating AOM.

The impact of AOM compliance and duration on weight loss is also unclear. In the real world, many patients discontinue AOM after reaching their desired weight loss goal. In the clinical trials for naltrexone–bupropion (Contrave®), 42% to 49% of patients dropped out due to medication nonadherence or other reasons related to weight gain 14. In the 2‐year SEQUEL extension study for controlled‐release phentermine/TPM, 84% (568/676) of patients completed the study, while 16% of patients discontinued due to either nonadherence or unrelated reasons 15. In these studies, dropouts were attributed not only to medication nonadherence but also to nonadherence to lifestyle and behavioural changes. There is only one study to our knowledge comparing shorter versus longer duration AOM use. In that study, longer duration phentermine was associated with greater weight loss up to 2 years after initiation 16.

This study evaluated the impact of medication initiation, usage and duration on weight loss in outcomes in a commercial precision medicine weight loss programme that incorporates clinical visits, a mobile application (mHealth) and tailored nutritional programming.

## Methods

### Study design

This is a retrospective cohort study analysing the impact of AOM on weight reduction in adults participating in a comprehensive precision weight loss programme (Enara Health). Data were collected from the electronic health records of patients who enrolled and started a weight loss journey during the period from 1 March 2015 to 1 April 2017 (with follow‐up through 1 October 2018). Enara Health provided the study investigators with all relevant data following de‐identification of the entire dataset. The Hummingbird Institutional Review Board (Needham, MA, USA) approved the study protocol and deemed that the study met the requirements for exemption from the US Department of Health and Human Services regulations for the protection of human subjects (45 CFR 46.101[b][4]).

### Weight loss programme interventions

Enara Health is a medical company based in San Mateo, California, that offers a unique hybrid digital and in‐person weight loss programme. The Enara Health weight loss programme has been operating since March 2015. By combining mobile technology with healthcare visits, the programme provides patients with personalized physician‐driven medical weight loss programmes. Upon enrolment, patients are initially evaluated by a physician or physician's assistant who determines the severity of obesity and the patient's readiness to change and performs a full metabolic workup and examination. Patients engage in one‐on‐one meetings with a registered dietician on a weekly basis during the first 3 months, once every other week during the next 3 months and then once monthly for 6 months. Patients also see their physician or physician assistant on average once a month or the first 3 months, and then every 3 months thereafter. Patients may conduct their visits either in‐person or via face‐to‐face video communication. There is a monthly fee of $50 for patients with insurance (most insurances accepted) or $300 without insurance.

Between visits, patients utilize an mHealth application, which delivers tailored educational content, messages of encouragement and support, nutritional and behavioural feedback, a review of meals and feedback by a registered dietician, as well as exercise suggestions, planning and encouragement by a fitness trainer. Patients may choose between an intense, rapid weight loss programme (daily caloric intake: 800–1,000 kcal) or a non‐intensive weight loss programme that emphasizes non‐processed foods but does not encourage calorie counting. Both options encourage whole, unprocessed, low glycaemic foods. Although all participants are assigned a target exercise goal of 150 min week^−1^, a personal exercise coach determines the duration of exercise based on the patient's physical ability. Many patients utilize activity trackers such as Fitbit® or Apple Watch® to monitor weekly exercise.

Where appropriate, patients are prescribed medications to assist with weight loss. Prescriptions are driven by the patient's request after attending an educational visit on weight loss medications. AOMs are prescribed in accordance with the US Food and Drug Administration‐labelled recommendations; however, in the event of non‐coverage, generic equivalents are prescribed, and any off‐label usage is discussed with the patient. The choice of medication is ultimately made by the patients and their physicians.

Prescribed weight loss medications include lorcaserin, phendimetrazine, diethylpropion, phentermine/TPM, liraglutide and bupropion/naltrexone. Some patients may be prescribed more than one AOM. Routine follow‐up visits with the provider are conducted to ensure that the patient is responding positively to the medication and is not experiencing adverse effects. All patients are started on the lowest dosage of medications, while dose adjustments are made on an individual basis as needed to achieve the desired outcomes.

### Study cohort selection

Patients enrolled in the Enara Health weight loss programme during the study period, who were aged ≥18 years with a BMI ≥30 kg m^−2^ at enrolment, were eligible for inclusion. Patients who became pregnant during the study period were excluded from the analyses.

### Assignment of anti‐obesity medication subgroups

Overall, participants were matched into a cohort so long as their start date indicated that they could have participated for the study duration regardless of their engagement status at that time point. Subgroups were assigned by AOM usage (users versus non‐users), timing of AOM initiation (early versus delayed) and treatment duration (short term versus long term) and were defined as follows.

#### Anti‐obesity medication users versus anti‐obesity medication non‐users

A patient was defined as an AOM user so long as the electronic health records documented medication use for a period exceeding 1 month. All other patients were considered to be non‐users. A 1‐month cut‐off was deemed necessary because many patients who had been prescribed AOM had not initiated treatment due to coverage‐related or cost‐related reasons, and compliance was only recorded on a monthly basis in the medical record.

#### Early starters versus delayed starters

For patients who used more than one AOM, the status of initiating an AOM was assigned based on the date of the first AOM prescribed. Early starters were defined as patients who initiated an AOM within 2 months of the date of programme enrolment, whereas delayed starters initiated an AOM >2 months after the date of programme enrolment.

#### Short duration versus long duration anti‐obesity medication

Anti‐obesity medication treatment duration represented the period commencing on the date of the first recorded AOM prescription and ending on the date of cessation of all AOMs prescribed. Patients who received ≥1 AOM for a total duration of 1–6 months were considered to have short duration AOM treatment, whereas patients who received ≥1 AOM for >6 months had long duration.

### Study data collection

Enara Health collected demographic and clinical data for all study patients at the initial clinic visit. Height, body weight, blood pressure, pulse and body composition were measured at the first appointment to establish baseline values and then rechecked in the clinic at every in‐person appointment thereafter. Body weight was collected daily via the patient's home scale, which connected directly to Enara Health's provider dashboard. Medical providers documented AOM compliance on a monthly basis.

### Study endpoints

The intention‐to‐treat (ITT) duration and time endpoint for the primary analysis were 72 weeks. Primary endpoints included weight change at 72 weeks from baseline and the proportion of patients who lost ≥5%, ≥10% and ≥15% of their baseline body weight. Secondary outcomes included weight change from baseline and categorical weight loss at 24 and 48 weeks.

### Statistical analysis

In the primary analysis of weight loss in AOM users and non‐users, an ITT analysis was performed. All individuals who started and enrolled in a weight loss programme were included in this analysis. If missing, imputed data were carried through from the last measured observation to the study endpoint. A ‘completers’ analysis was conducted to evaluate the subgroups characterized by AOM initiation and duration due to the potential bias associated with missing data carried forward and given that AOM status imputations with statistical modelling were underpowered. The completers analysis included all patients who had data on weight loss and AOM status at 72 weeks.

At each time endpoint of interest, change in baseline body weight was expressed as the mean percentage change and the mean (± standard deviation) absolute difference in kilograms of body weight. The proportions of patients achieving ≥5%, ≥10% and ≥15% weight loss in each subgroup were also calculated. Differences in weight change and in the proportions attaining each percentage category of weight loss were compared between the subgroups using a *t*‐test for continuous data and a Chi‐square test for categorical data.

All statistical analyses were performed using Python 3.6. Statistical significance was defined as *P* < 0.05.

## Results

### Selection of study cohort

**Figure 1:**
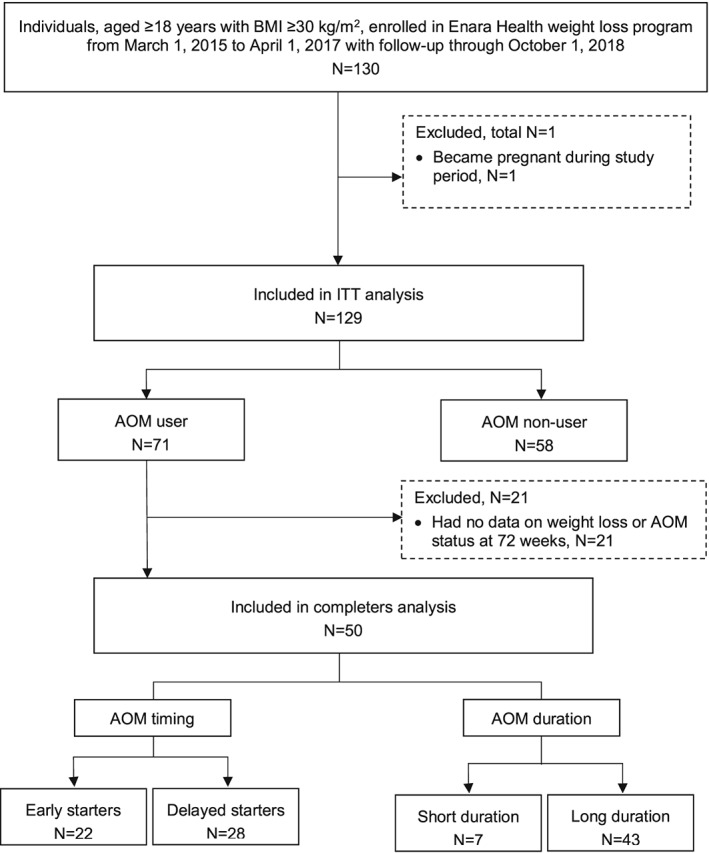
Study cohort selection flow diagram. AOM, anti‐obesity medication; BMI, body mass index; ITT, intent‐to‐treat.

Figure 1 shows the disposition and selection of the final study cohort and AOM subgroups. Data were obtained for 130 patients enrolled in the Enara Health weight loss programme during the study period, of whom 129 (99.2%) met the study inclusion criteria and had valid data. Of the 129 participants, 71 (55%) AOM users were included in the subgroup (completers) analyses.

### Baseline patient characteristics

Table 1 presents the baseline characteristics of the total study cohort (ITT *N* = 129) and subgroups by AOM usage status. The mean age was 45 ± 14 years. The mean weight and mean BMI were 103 ± 23 kg and 37 ± 6 kg m^−2^, respectively. Women comprised approximately two‐thirds (67%) of the cohort. Compared with the non‐users (*N* = 58) at baseline, AOM users on average were significantly heavier (107.0 ± 22.0 vs. 97.0 ± 20.0 kg) and featured a higher proportion of men (41% vs. 24%), increased BMI (38.0 ± 6.0 vs. 35.0 ± 5.0 kg m^−2^) and lower high‐density lipoprotein cholesterol level (51.0 ± 11.7 vs. 57.0 ± 14.4 mg dL^−1^) (all differences, *P* < 0.05). Seventy‐seven per cent of AOM users and 71% of non‐users choose to do the intensive weight loss programme. The difference between the two cohorts was not statistically significant (*P* = 0.356).

**Table 1:** Participants' baseline characteristics

	All users (n = 129)	Non‐medication users (n = 58)	Medication users (n = 71)
Age (years)	45 ± 14	46 ± 14	44 ± 12
Baseline weight (kg)*	103 ± 23	97 ± 20	107 ± 22
BMI (kg m^−2^)*	37 ± 6	35 ± 5	38 ± 6
Gender (male), *n* (%)*	43 (33%)	14 (24%)	29 (41%)
Gender (female), *n* (%)*	86 (67%)	44 (76%)	42 (59%)
HbA1C (%)	5.70 ± 0.51 (*n* = 105)	5.63 ± 0.31 (*n* = 48)	5.75 ± 0.62 (*n* = 57)
Total cholesterol (mg dL^−1^)	200.38 ± 38.55 (*n* = 113)	205.52 ± 36.56 (*n* = 52)	196.0 ± 39.65 (*n* = 61)
Triglycerides (mg dL^−1^)	131.25 ± 63.92 (*n* = 114)	127.49 ± 56.38 (*n* = 53)	134.53 ± 69.67 (*n* = 61)
LDL cholesterol (mg dL^−1^)	120.09 ± 33.20 (*n* = 112)	123.58 ± 31.46 (*n* = 52)	117.07 ± 34.36 (*n* = 60)
HDL cholesterol (mg dL^−1^)*	53.71 ± 13.33 (*n* = 112)	56.98 ± 14.39 (*n* = 51)	50.98 ± 11.69 (*n* = 61)
Glucose (mg dL^−1^)	98.0 ± 14.49 (*n* = 100)	96.48 ± 9.73 (*n* = 43)	99.14 ± 17.15 (*n* = 57)

Values shown are *n* (%) or means ± standard deviation.

* Categories differ significantly from each other at *P* < 0.05.

BMI, body mass index; HbA1c, haemoglobin A1c; HDL, high‐density lipoprotein; LDL, low‐density lipoprotein.

Of the 71 AOM users, 50 (70.4%) had a weigh‐in at 72 weeks and were considered completers (Table 2). Medication utilization among the AOM user subgroup was as follows: phentermine/TPM extended‐release (*n* = 44, 62%), phentermine (*n* = 17, 24%), liraglutide (*n* = 12, 17%), bupropion/naltrexone (*n* = 9, 13%) and TPM (*n* = 5, 7%). Twenty‐two per cent (*n* = 16) of AOM users used a combination of two AOMs. Of the 50 completers, 22 (44%) were early AOM starters and 28 (56%) were delayed AOM starters. With respect to treatment duration, 43 of the 50 completers (86%) had been treated for >6 months; seven (14%) had been treated for ≤6 months. While the baseline characteristics did not differ significantly between early and delayed AOM starters, completers who had been treated for a long duration had a significantly higher mean BMI compared with those treated for a short duration (39 ± 6.8 vs. 33 ± 3.2 kg m^−2^; *P* < 0.05).

**Table 2:** Medication users' baseline characteristics (completers only)

	All medication users (n = 50)	Early starters (n = 22)	Delayed starters (n = 28)	Short duration (n = 7)	Long duration (n = 43)
Age (years)	45 ± 13.04	45 ± 12.2	45 ± 13.66	44 ± 14.93	45 ± 12.69
Baseline weight (kg)	107 ± 24.9	114 ± 30.54	102 ± 17.49	92 ± 11.06	110 ± 25.68
BMI (kg m^−2^)	38 ± 6.7	39 ± 7.63*	37 ± 5.59*	33 ± 3.24	39 ± 6.78
Gender (male), *n* (%)	20 (40%)	6 (27%)	14 (50%)	4 (57%)	16 (37%)
Gender (female), *n* (%)	30 (60%)	16 (73%)	14 (50%)	3 (43%)	27 (63%)
HbA1C (%)	5.82 ± 0.41 (*n* = 40)	5.84 ± 0.48 (*n* = 19)	5.81 ± 0.36 (*n* = 21)	5.82 ± 0.33 (*n* = 6)	5.83 ± 0.43 (*n* = 34)
Total cholesterol (mg dL^−1^)	199.67 ± 37.04 (*n* = 43)	198.72 ± 34.17 (*n* = 18)	200.36 ± 38.96 (*n* = 25)	213.86 ± 56.23 (*n* = 7)	196.92 ± 31.26 (*n* = 36)
Triglycerides (mg dL^−1^)	142.82 ± 69.31 (*n* = 43)	154.44 ± 76.96 (*n* = 18)	134.46 ± 61.90 (*n* = 25)	143.86 ± 83.98 (*n* = 7)	142.62 ± 66.08 (*n* = 36)
LDL cholesterol (mg dL^−1^)	118.86 ± 31.95 (*n* = 42)	114.22 ± 27.52 (*n* = 18)	122.33 ± 34.50 (*n* = 24)	137.0 ± 49.91 (*n* = 7)	115.23 ± 25.46 (*n* = 35)
HDL cholesterol (mg dL^−1^)	51.46 ± 11.76 (*n* = 43)	54.18 ± 12.69 (*n* = 18)	49.52 ± 10.62 (*n* = 25)	45.29 ± 13.18 (*n* = 7)	52.67 ± 11.07 (*n* = 36)
Glucose (mg dL^−1^)	99.93 ± 16.00 (*n* = 41)	95.74 ± 12.63 (*n* = 19)	103.54 ± 17.65 (*n* = 22)	101.0 ± 6.07 (*n* = 5)	99.78 ± 16.92 (*n* = 36)

Values shown are *n* (%) or means ± standard deviation.

* Categories differ significantly from each other at *P* < 0.05.

BMI, body mass index; HbA1c, haemoglobin A1c; HDL, high‐density lipoprotein; LDL, low‐density lipoprotein.

### Primary endpoint analysis

#### Comparison of changes in baseline weight between anti‐obesity medication users versus non‐users at week 72 (intention‐to‐treat cohort)

**Figure 2:**
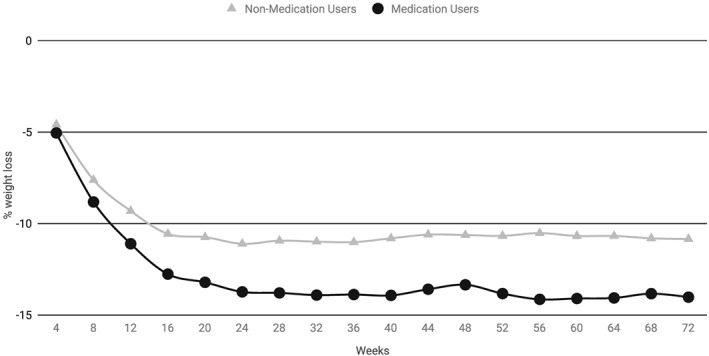
Differences in mean percentage weight reduction over 72 weeks between anti‐obesity medication users and non‐users. Estimated mean percentage reduction in baseline weight over 72 weeks in the intention‐to‐treat cohort (71 anti‐obesity medication users and 58 non‐users). P‐values for differences between the subgroups (per t‐test) at week 24, week 48 and week 72 are shown in Table 3.

**Figure 3:**
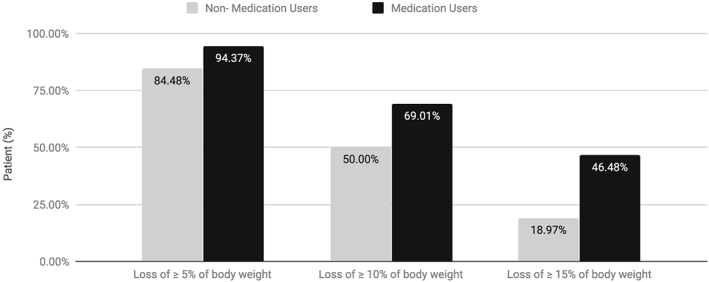
Categorical (5%, 10% and 15%) weight loss at week 72 in anti‐obesity medication users versus non‐users. Differences in the proportions of patients with 10% and 15% categorical weight loss between anti‐obesity medication users and non‐users were statistically significant (P < 0.05, per Chi‐square tests) (Table 3).

At week 72, AOM users (*N* = 71) achieved significantly greater mean percentage and absolute reductions in baseline weight than non‐users (*N* = 58) (Figures 2 and 3 and Table 3). On average, the baseline weight was decreased by 14.0 ± 6.2% in AOM users compared with 10.9 ± 6.8% in non‐users (*P* = 0.008). The mean absolute weight loss was 15.0 ± 7.2 kg for AOM users and 10.4 ± 6.9 kg for non‐users (*P* < 0.001). Compared with non‐users, a markedly higher proportion of AOM users attained weight reductions ≥10% of the baseline (69% vs. 50%; *P* = 0.03), as well as ≥15% of the baseline (45.1% vs. 19.0%, respectively; *P* < 0.001).

**Table 3:** Changes in primary endpoints

	Non‐medication users (n = 58)	Medication users (n = 71)	P‐value
Change in weight (%)			
Week 24	−11.12 ± 5.15	−13.76 ± 6.05	0.009*
Week 48	−10.64 ± 6.13	−13.36 ± 6.32	0.016*
Week 72	−10.86 ± 6.84	−14.04 ± 6.22	0.008*
Change in weight (kg)			
Week 24	−10.72 ± 5.90	−14.74 ± 6.94	0.001*
Week 48	−10.13 ± 6.35	−14.43 ± 7.32	0.001*
Week 72	−10.35 ± 6.90	−14.99 ± 7.19	<0.001*
Loss of ≥5% of body weight (%)			
Week 24	93.10%	95.77%	0.51
Week 48	84.48%	92.96%	0.12
Week 72	84.48%	94.29%	0.06
Loss of ≥10% of body weight (%)			
Week 24	53.45%	69.01%	0.07
Week 48	51.72%	59.15%	0.31
Week 72	50.00%	69.01%	0.03*
Loss of ≥15% of body weight (%)			
Week 24	22.41%	40.85%	0.03*
Week 48	17.24%	39.44%	0.01*
Week 72	18.97%	45.07%	<0.001*

Values shown are *n* (%) or means ± standard deviation for the intention‐to‐treat population (*N* = 129).

* Categories differ significantly from each other at *P* < 0.05.

#### Subgroup comparisons of changes in baseline weight at week 72 (completers)

**Figure 4:**
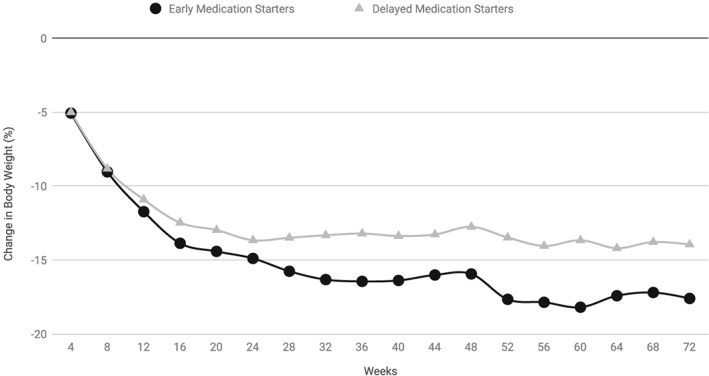
Differences in mean per cent weight reduction over 72 weeks between anti‐obesity medication early starters versus delayed starters. Estimated mean percentage reduction in baseline weight over 72 weeks in anti‐obesity medication completers (N = 50) with early start (N = 22) and delayed start (N = 28) of treatment. P‐values (per t‐tests) for differences between the subgroups at week 24, week 48 and week 72 are shown in Table 4.

At week 72, AOM early starters (*N* = 22) achieved significantly greater mean percentage and absolute reductions in baseline weight than delayed starters (*N* = 28) (Figure 4 and Table 4). On average, the baseline weight was decreased by 17.6 ± 5.3% in early starters compared with 14.0 ± 5.5% in delayed starters (*P* = 0.024). The mean absolute weight loss was 19.7 ± 6.7 kg for early starters and 14.1 ± 5.6 kg for delayed starters (*P* = 0.003). A markedly higher proportion of early starters attained weight reductions ≥15% of the baseline compared with delayed starters (72.3% vs. 42.9%, respectively; *P* = 0.03).

**Table 4:** Changes in primary endpoints among medication users

	Early starters (n = 22)	Delayed starters (n = 28)	P‐value	Short duration (n = 7)	Long duration (n = 43)	P‐value
Change in weight (%)						
Week 24	−14.90 ± 6.22	−13.68 ± 5.73	0.425	−14.98 ± 6.03	−14.15 ± 6.01	0.688
Week 48	−15.95 ± 6.52	−12.76 ± 4.58	0.047*	−13.39 ± 3.75	−14.40 ± 6.09	0.536
Week 72*	−17.6 ± 5.30	−13.95 ± 5.48	0.024*	−12.67 ± 4.21	−16.03 ± 5.77	0.114
Change in weight (kg)						
Week 24	−16.12 ± 6.75	−14.49 ± 7.15	0.361	−15.58 ± 7.47	−15.27 ± 6.88	0.9
Week 48	−17.39 ± 7.12	−13.22 ± 5.45	0.021*	−13.29 ± 5.10	−15.52 ± 6.81	0.298
Week 72*	−19.67 ± 6.66	−14.07 ± 5.59	0.003*	−11.82 ± 4.16	−17.30 ± 6.71	0.019*
Loss of ≥5% of body weight (%)						
Week 24	96.97%	96.77%	0.96	91.67%	98.08%	0.25
Week 48	100.00%	96.67%	0.35	100.00%	97.87%	0.66
Week 72*	100.00%	96.43%	0.373	100.00%	97.67%	0.68
Loss of ≥10% of body weight (%)						
Week 24	75.76%	67.74%	0.48	75.00%	71.15%	0.79
Week 48	80.77%	56.67%	0.05*	77.78%	65.96%	0.49
Week 72*	90.91%	75.00%	0.15	71.43%	83.72%	0.43
Loss of ≥15% of body weight (%)						
Week 24	48.48%	41.94%	0.6	58.33%	42.31%	0.31
Week 48	53.85%	33.33%	0.12	44.44%	42.55%	0.92
Week 72*	72.73%	42.86%	0.03*	28.57%	58.14%	0.15

Values shown are *n* (%) or means ± standard deviation for completers analysis (*N* = 50) in the medication subgroup.

* Categories differ significantly from each other at *P* < 0.05.

The mean percentage reduction in baseline weight at 72 weeks was numerically greater for completers treated with AOM for a long duration (*N* = 43) than for those treated for a short duration (*N* = 7) (−16.0 ± 5.7% vs. −12.7 ± 4.2%, respectively), albeit not statistically different (Table 4). The mean absolute weight loss, however, was significantly increased with long treatment duration (17.3 ± 6.7 vs. 11.8 ± 4.2 kg; *P* = 0.019). The proportions of patients achieving categorical percentage losses did not differ significantly between the subgroups.

### Analysis of secondary endpoints

#### Comparison of changes in baseline weight between anti‐obesity medication users versus non‐users at weeks 24 and 48 (intention‐to‐treat cohort)

At both weeks 24 and 48, AOM users achieved significantly greater mean percentage and mean absolute reductions in baseline weight compared with non‐users. On average, baseline weight was reduced by 13.8% in AOM users and by 11.1% in non‐users at week 24 (*P* = 0.009) and by 13.4% and 10.6% in the respective subgroups at week 48 (*P* = 0.016). At week 24, the mean absolute weight loss was 14.7 ± 6.9 kg for AOM users and 10.7 ± 5.9 kg for non‐users (*P* = 0.001). At week 48, the mean absolute weight loss was 14.4 ± 7.3 kg for AOM users and 10.1 ± 6.4 kg for non‐users (*P* = 0.001). Significantly higher proportions of AOM users lost ≥15% of their baseline weight than non‐users at both week 24 (40.9% vs. 22.4%; *P* = 0.03) and week 48 (39.4% vs. 17.2%; *P* = 0.01).

#### Subgroup comparisons of changes in baseline weight at weeks 24 and 48 (completers)

At week 24, early starters (*N* = 22) exhibited numerically higher mean percentage and absolute reductions in baseline weight compared with delayed starters (*N* = 28), albeit the differences were not significant (Table 4). The proportions of patients achieving categorical percentage losses did not differ significantly between the subgroups. At week 48, however, early starters achieved marked percentage and absolute reductions in baseline weight compared with delayed starters. On average, the baseline weight was decreased by 16% in early starters compared with 12.8% in delayed starters (*P* = 0.047). The mean absolute weight loss was 17.4 ± 7.1 kg for early starters and 13.2 ± 5.5 kg for delayed starters (*P* = 0.021). A higher proportion of early starters attained weight reductions ≥10% of the baseline at week 48 compared with delayed starters (80.8% vs. 56.7%, respectively; *P* = 0.05).

At both weeks 24 and 48, the AOM subgroups categorized by treatment duration did not differ significantly with respect to the magnitude of changes in baseline weight or the proportions achieving categorical percentages of weight loss (Table 4).

## Discussion

This study supports that AOM has an incremental impact on weight loss efforts with lifestyle modification 17, 18. The addition of AOM by patients was associated with a greater mean percentage reduction in weight (14.2% vs. 10.8%) and a higher proportion achieving 15% weight loss (45.1% vs. 19.0%). This was not a randomized clinical trial and was not restricted to one medication; rather, it is a study of the real‐world application of obesity medications where a clinician works to find the optimal medications for a patient based on past medical history, contraindications, coverage and preference. In a handful of studies, higher initial BMI is associated with less weight loss after surgical interventions 19, 20. However, the impact of initial BMI on AOM efficacy has not been studied. In our study, the association of AOM with greater per cent weight loss in patients who were heavier at baseline (and had greater disease burden) suggests that BMI‐adjusted differences are likely to be even greater.

More importantly, this study adds to the growing body of indirect evidence elucidated in the introduction showing that medication timing and duration may perhaps play a significant role in outcomes. Patient who began an AOM at the onset of their behavioural modification achieved an impressive 17.6% weight loss at 72 weeks; 72% of this subset achieved >15% weight loss. We believe that patients who begin an AOM at onset and continue to engage in behaviour modification may experience less metabolic adaptation as they lose weight 6, 21, 22, 23. A prospective study examining early versus delayed medication initiation on metabolic adaptation parameters would be very informative. If newer obesity medications are found to play a role in temporizing the onset of metabolic adaptation, then earlier introduction during a patient's weight loss journey may help sustain outcomes.

Our findings differ from studies demonstrating additional weight loss when TPM or liraglutide were added after low‐calorie‐induced weight loss 7, 9 compared with studies initiating therapy at programme onset 8, 10. The difference in weight loss observed may likely be explained by varying dietary and behavioural protocols rather than delayed onset of medication. Alternatively, in real‐world application, early AOM initiation may reinforce dietary and behavioural plans and encourages patients to engage longer.

While medication duration was not found to be significant, greater weight loss and categorical weight loss were observed in subgroup receiving treatment for a long duration. Additionally, the short duration subgroup displayed some evidence of weight recidivism at week 72. This correlates with studies showing that when AOM is stopped, weight increases gradually to the level seen with lifestyle changes alone 24.

The level of weight loss observed in this study is greater than that observed in AOM randomized controlled trials; however, the difference between AOM user and non‐users subgroups observed in the present study was similar to that reported in the literature 17, 18, 25. The varying weight loss outcomes between studies is an added indication that dietary, behavioural and educational components of lifestyle modification interventions may enhance outcomes.

Study strengths include a 72‐week treatment duration, which allowed for exploration of the downstream impact of AOMs, and the high completer rate (71%) for patients electing treatment with AOM. Potential limitations include the retrospective nature, small sample sizes in the completer subgroups and the gender and BMI differences between AOM users and non‐users in the ITT analysis. Differences in starting BMI and weight between AOM user and non‐users were adjusted by using per cent weight loss rather than absolute weight loss as the primary endpoint. These differences are also informatory as it suggests that in real‐world settings, male patients and patients who have greater obesity burden are more open to using AOM.

Precision medicine calls for the use of genomic and metabolic data to personalize medication delivery 26. In the context of obesity, the timing and duration of medication against the backdrop of social, behavioural, nutritional and environmental factors become additional precision elements 26. Obesity is complicated disease that will require multimodal lifestyle modification interventions, and our understanding of why some individuals respond well to certain treatments while others do not is limited 27. While current focus is on how genomic, behavioural, biological, environmental and psychosocial factors impact treatment, we must also consider how different treatment(s) interact and impact one another. The different combinations and order in which therapies are delivered present an opportunity to optimize outcomes.

## Funding

This work was funded by Enara Health.

## Conflict of Interest Statement

Rojin Safavi was not compensated for this study nor does she have any conflicts of interest. The remaining authors all work for Enara Health and do not have any other declarations.
